# Superoxide dismutase 1 mediates adaptation to the tumor microenvironment of glioma cells via mammalian target of rapamycin complex 1

**DOI:** 10.1038/s41420-024-02145-6

**Published:** 2024-08-26

**Authors:** Sven König, Florian Strassheimer, Nadja I. Brandner, Jan-Hendrik Schröder, Hans Urban, Leander F. Harwart, Stephanie Hehlgans, Joachim P. Steinbach, Michael W. Ronellenfitsch, Anna-Luisa Luger

**Affiliations:** 1 Goethe University Frankfurt, University Hospital, Dr. Senckenberg Institute of Neurooncology, Frankfurt am Main, Germany; 2 German Cancer Consortium (DKTK), Partner Site Frankfurt/Mainz, a partnership between DKFZ and University Hospital Frankfurt, Frankfurt am Main, Germany; 3 Goethe University Frankfurt, Frankfurt Cancer Institute (FCI), Frankfurt am Main, Germany; 4 Goethe University Frankfurt, University Hospital, University Cancer Center (UCT), Frankfurt am Main, Germany; 5 Goethe University Frankfurt, University Hospital, Department of Radiotherapy and Oncology, Frankfurt am Main, Germany

**Keywords:** CNS cancer, Cancer microenvironment, Cancer therapeutic resistance

## Abstract

In glioblastoma (GB) cells oxidative stress is induced by both, conditions of the tumor microenvironment as well as by therapeutic interventions. Upregulation of superoxide dismutase 1 (SOD1), a key enzyme for oxidative defense and downstream target of mammalian target of rapamycin complex 1 (mTORC1) is a candidate mechanism to sustain survival and proliferation of tumor cells. SOD1 was inhibited by shRNA mediated gene suppression, CRISPR/Cas9 knockout and pharmacological inhibition in human (primary) GB cells. SOD1 activity was determined by SOD1/2 activity assay. ROS levels, cell death and the NADPH/NADP-ratio were measured under normal and starvation conditions. To study the mTORC1-SOD1 axis, mTORC1 activated *TSC2* knockdown cells (TSC2sh) were analyzed. Genetic and pharmacological inhibition of *SOD1* correlated with decreased SOD1 activity, increased ROS and enhanced the sensitivity of glioma cells towards starvation- and hypoxia-induced cell death. This was accompanied by a decreased NADPH/NADP-ratio. Furthermore, combination therapy of SOD1 and mTORC1 inhibition partially rescued the protective effect of mTORC1 inhibitor monotherapy. SOD1 mediates adaptation of GB cells to stress conditions in the tumor microenvironment in a mTORC1-dependent manner. Moreover, SOD1 activation contributes to the cell death resistance conferred by mTORC1 inhibitors under hypoxic conditions.

## Introduction

Glioblastoma (GB) is an incurable brain cancer with a median survival below two years [1]. Standard first line treatment involves tumor resection followed by adjuvant radiochemotherapy with temozolomide and the application of tumor-treating fields (TTFields) [2, 3]. In almost all GB patients, the disease progresses within less than one year [1]. After tumor progression no standardized treatment has been established so far [1]. Characterized by their aggressive nature and often dismal prognosis, these tumors demand a comprehensive exploration of the underlying molecular mechanisms that drive pathogenesis and progression [1].

The microenvironment of GB is characterized by nutrient deprivation and hypoxia due to uncontrolled tumor growth promoting selection for the most resistant cell clones [4–9]. Both, microenvironmental conditions including hypoxia and nutrient deprivation as well as therapeutic interventions induce oxidative stress as a major challenge for tumor cells to sustain survival under these adverse conditions [6, 8, 10].

Reactive oxygen species (ROS) homeostasis is required for the survival of normal cells and appropriate cell signaling, but is also involved in the development of cancer [11, 12]. Cancer cells are characterized by regional increased ROS e.g. generated by starvation conditions and/or by the activation of oncogenes and loss of tumor suppressor genes [13–15]. An important feature of tumor cells is the ability to tolerate these elevated ROS levels and ensure survival under adverse microenvironmental conditions [13, 15]. This is accomplished e.g. by activating the transcription factor Nuclear factor erythroid-2-related factor 2 (NRF2), which increases the expression of antioxidant enzymes such as superoxide dismutases (SODs), peroxiredoxins, catalase and glutathione peroxidases [10, 11, 13, 14, 16]. Moreover, mitophagy and the cofactors reduced glutathione and NADPH are fundamental for intact antioxidant defense mechanisms [11, 17]. SODs play a central role in catalyzing the dismutation of superoxide radicals into oxygen and hydrogen peroxide, thus acting as a primary line of defense against the harmful effects of excessive ROS [12, 18, 19]. Superoxide dismutase 1 (SOD1), a ubiquitous copper- and zinc-containing enzyme is localized in the cytoplasm and nucleus [20, 21]. SOD2, a manganese superoxide dismutase, is localized in mitochondria [21, 22] and SOD3 acts as the extracellular isoform [19, 21]. Due to its high antioxidant activity and its direct control over the cellular ROS levels, SOD1 plays a crucial role in enabling the survival of tumor cells in a hypoxic tumor environment [14, 21, 23].

Inhibition of the commonly altered signaling pathway downstream of mammalian target of rapamycin complex 1 (mTORC1) is a plausible therapeutic approach in cancer [24]. However, in a previous study we have shown that mTORC1 inhibition causes metabolic changes leading to a protection from hypoxia-induced cell death [25, 26] and potentially from temozolomide [27, 28]. Moreover, it is already known that vice versa EGFR or mTORC1 activation leads to sensitization to hypoxia-induced cell death [29, 30]. One possible explanation for the resistance of GBs to mTORC1-inhibitors could be the recently identified activation of SOD1 following mTORC1 inhibition [14]. According to previous studies [14, 16], human SOD1 is a downstream target of mTORC1 and is directly regulated by mTORC1 via phosphorylation at Thr40 [23] leading to a deactivation of SOD1 function [14, 23]. In starved tumor regions, mTORC1 is physiologically inhibited leading to a dephosphorylation and consecutive activation of SOD1 enhancing the cellular redox defense capacity [14].

Although it is already known that *SOD1* is upregulated in cancers [31–33], SOD1 function and its potential as therapeutic target have been poorly studied in glioma cells. Ling et al. [31] have shown that inhibition with the small molecule inhibitor of SOD1 LCS-1 mediates ROS-dependent cell death. Another promising small molecule inhibitor of SOD1 is ATN-224, which caused increased superoxide and hydrogen peroxide levels and leads to cell death in lung cancer cells [34]. This inhibitor has not yet been tested in glioma cells. By targeting SOD1, ATN-224 may disrupt the ROS-regulatory network within glioma cells, which could have profound implications for tumor behavior and therapeutic response [23]. By exploring mechanisms of action, potential synergies with mTORC1 inhibitors, and implications for glioma cell biology, we aim to highlight the potential benefits and challenges of targeting SOD1.

We report that genetic and pharmacological inhibition of SOD1 leads to sensitization to hypoxia-induced cell death and disruption of redox balance under conditions of the glioma microenvironment.

## Results

### Identification of SOD1 as a regulator of adaptation to the tumor microenvironment

**Fig. 1:**
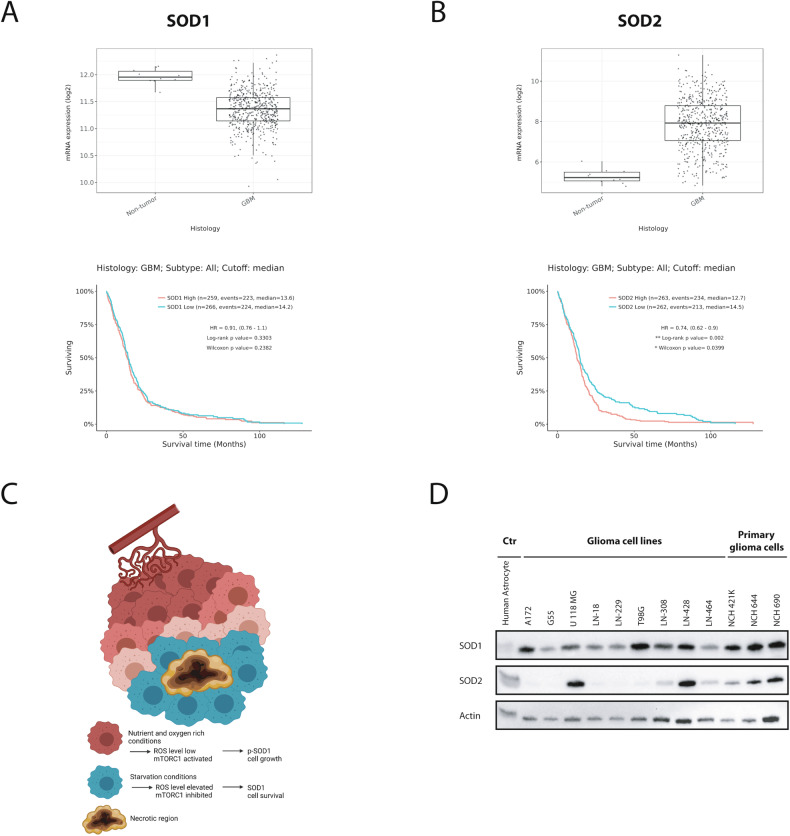
*SOD1* gene expression in human GBs. **A**, **B** Analysis of SOD1 and SOD2 expression and median overall survival in GBs using the TCGA GBM database via Gliovis [35]. **C** Physiological mTORC1 inhibition induced by hypoxia and nutrient deprivation causes a dephosphorylation and activation of SOD1 to potentially improve cell survival (Created with BioRender.com, modified after Tsang et al.). **D** Expression of SOD1 and SOD1 in human astrocytes, glioma cell lines and primary glioma cells in normoxia without nutrient deprivation were analyzed by immunoblot.

To analyze potential prognostic effects of SOD1/2 in GB patients, we analyzed publicly available databases for SOD1 and SOD2 expression in gliomas. Although a slight increase in SOD1 expression with WHO Grade has been suggested in gliomas [31], analysis of the TCGA GBM and Rembrandt databases did not show that SOD1 is upregulated in GBs (Fig. 1A) or increases with WHO Grade (Supplementary Fig. 1) [35]. SOD1 expression has furthermore no effect on overall survival in GBs (Fig. 1A) [35]. In contrast, SOD2 expression is increased in GBs and a high expression correlates with an inferior survival of GB patients (Fig. 1B, Supplementary Fig. 1) [35]. Although the expression of SOD1 in GBs was not significantly altered, recent publications indicate a regulation of SOD1 activity by the tumor microenvironment [14]. In starved tumor regions mTORC1 is physiologically inhibited leading to a dephosphorylation and activation of SOD1 enhancing the cellular redox defense capacity (Fig. 1C). These effects cannot be detected by conventional expression analyses. However, in order to identify representative glioma cell lines for further experiments the basal expression of SOD1 and 2 was examined in different human (primary) GB cell lines and human astrocytes (Fig. 1D).

For further studies LN-229, T98G and NCH690 cells were selected after consideration of a high SOD1 expression with low SOD2 expression and adherent growth to ensure comparability of the results.

### Gene suppression of *SOD1* increases cellular ROS levels and sensitizes human GB cells to nutrient deprivation and hypoxia

**Fig. 2:**
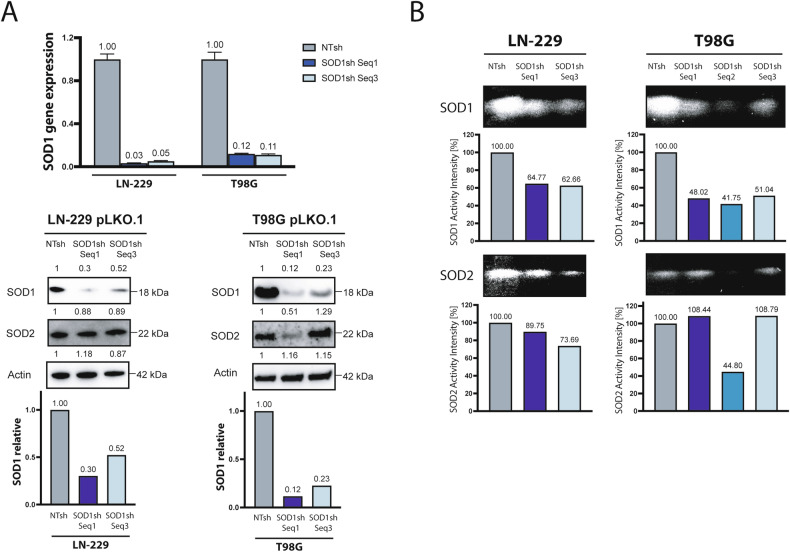
SOD1 knockdown in human GB cells. **A** LN-229 and T98G SOD1sh and control cells (non-targeting sequence, NTsh) were analyzed by qPCR. *SOD1* gene suppression was confirmed. Values are normalized to 18 S as well as *SDHA* housekeeping gene expression (*n* = 3, mean +/- SD). Western blot analysis shows a *SOD1* knockdown efficiency of up to 90%. *SOD1* knockdown efficiency was tested via western blot analysis. **B** Specific SOD1 and SOD2 enzyme activity assay of LN-229 and T98G SOD1sh and control cells.

To investigate the effects of SOD1 activity in GB cells under starvation including hypoxia and nutrient deprivation, cells with gene suppression of *SOD1* were generated. QPCR and immunoblot confirmed stable gene suppression of *SOD1* (SOD1sh) compared to control cells (NTsh) (Fig. 2A). SOD1 and SOD2 enzyme activity assay (Fig. 2B) confirmed a significant decrease in SOD1 enzyme activity compared to control cells (NTsh). For further studies the two knockdown sequences (from here on referred to as SOD1sh Seq1 and Seq3) with no relevant suppression of SOD2 activity were used.

**Fig. 3:**
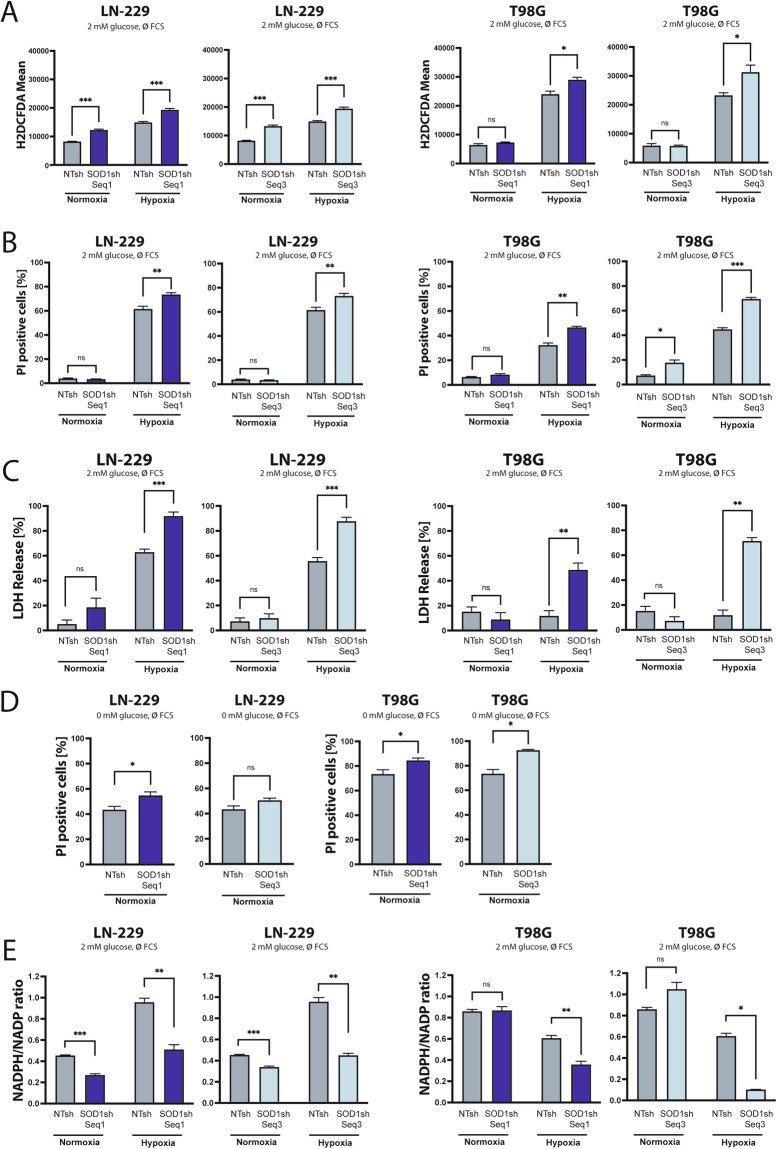
*SOD1* knockdown increases ROS levels and sensitizes human GB cells to nutrient deprivation and hypoxia. **A** LN-229 and T98G SOD1sh and NTsh cells were treated in serumfree DMEM containing 2 mM glucose in normoxia or hypoxia (0.1%) for 16 h (LN-229) or 13 h (T98G). Cells were analyzed by H2DCFDA and PI staining. At this timepoint no relevant cell death was observed (data not shown) and only PI negative ( = living) cells were quantified for H2DCFDA FACS analysis (*n* = 4, mean ± SEM, student’s t-test, *p < 0.05, **p < 0.01, ***p < 0.001). **B** LN-229 and T98G SOD1sh and NTsh cells were treated in serumfree DMEM containing 2 mM glucose in normoxia or hypoxia (0.1%) for 20 h (LN-229) and 16 h (T98G). Cell death was analyzed by PI staining and quantified by flow cytometry (*n* = 4, mean ± SEM, student’s t-test, *p < 0.05, **p < 0.01, ***p < 0.001). **C** LN-229 and T98G SOD1sh and NTsh cells were treated in serumfree DMEM containing 2 mM glucose in normoxia or hypoxia (0.1%) for 34 h (LN-229) and 25 h (T98G). Cell death was analyzed by LDH release assay (*n* = 4, mean ± SEM, student’s t-test, *p < 0.05, **p < 0.01, ***p < 0.001). **D** LN-229 SOD1sh and T98G SOD1sh and NTsh cells were treated in serum- and glucosefree DMEM for 20 h (LN-229) and 21 h (T98G) in normoxia. Cell death was analyzed by PI staining and flow cytometry (*n* = 4, mean ± SEM, student’s t-test, *p < 0.05, **p < 0.01, ***p < 0.001). **E** LN-229 SOD1sh and T98G SOD1sh cells were treated in serumfree DMEM containing 2 mM glucose in normoxia or hypoxia (0.1%) for 14 h. After 14 h NADP-NADPH-Glo-Assay was performed and the NADPH/NADP ratio was determined (*n* = 3, mean ± SEM, student’s t-test, *p < 0.05, **p < 0.01, ***p < 0.001).

SOD1sh cells were characterized by higher ROS levels under nutrient deprivation and hypoxia (Fig. 3A). This effect was accompanied by an enhanced sensitivity to hypoxia-induced cell death in PI FACS analysis and LDH release assay (Fig. 3B, C). Additionally, *SOD1* knockdown cells were sensitized to nutrient deprivation induced cell death (Fig. 3D). In line with a reduced redox capacity gene suppression of *SOD1* resulted in a decreased NADPH/NADP-ratio, especially under hypoxic conditions (Fig. 3E).

### Primary glioblastoma *SOD1* knockdown cells and *SOD1* knockout cells are sensitized to hypoxia and characterized by reduced clonogenic survival

**Fig. 4:**
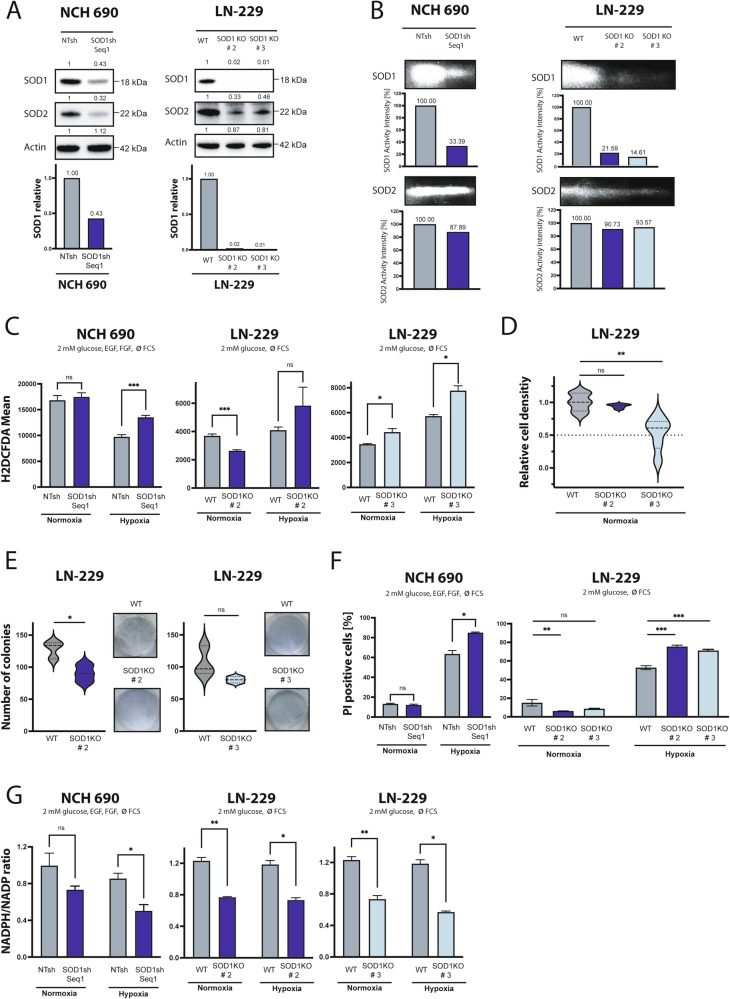
*SOD1* knockdown in human primary GB cells and *SOD1* KO in GB cells increases ROS levels, sensitizes to hypoxia-induced cell death and reduces clonogenic survival. **A** Lentiviral *SOD1* knockdown was performed in human primary GB cell lines NCH690 and a CRISPR/Cas9 *SOD1* knockout (KO) was performed in human GB cells LN-229. *SOD1* knockdown/-out was analyzed by immunoblot. **B** SOD1 and SOD2 enzyme activity assay showed a significant decrease of the SOD1 enzyme activity after *SOD1* knockdown in NCH 690 cells and LN-229 *SOD1* KO cells. **C** NCH 690 SOD1sh cells were treated in serumfree DMEM containing 2 mM glucose, 20 ng/ml human recombinant basic fibroblast (bFEF) and 20 ng/ml epidermal growth factor (EGF) in normoxia or hypoxia (0.1%) for 18 h. LN-229 *SOD1* knockout cells were treated in serumfree DMEM containing 2 mM glucose in normoxia or hypoxia (0.1%) for 16 h (# 2) or 19 h (# 3). Cells were analyzed by H2DCFDA and PI staining. At this timepoint no relevant cell death was observed (data not shown) and only PI negative ( = living) cells were quantified for H2DCFDA FACS analysis (*n* = 4, mean ± SEM, student’s t-test, *p < 0.05, **p < 0.01, ***p < 0.001). **D** LN-229 wt and *SOD1* KO cells were seeded in 96-well-plates in DMEM in normoxia for 96 h. Cell density was analyzed by CV staining (*n* = 3, mean, student’s t-test, *p < 0.05, **p < 0.01). **E** LN-229 WT and *SOD1* KO cells were seeded (500 cells per well) in 6-well-plates. Medium was changed after 24 h and cell growth of the clones was stopped via CV staining after 6 days (*n* = 3, mean, student’s t-test, *p < 0.05). **F** NCH 690 SOD1sh cells were treated in serumfree DMEM containing 2 mM glucose, 20 ng/ml human recombinant basic fibroblast (bFEF) and 20 ng/ml epidermal growth factor (EGF) in normoxia or hypoxia (0.1%) for 26 h. LN-229 *SOD1* KO cells were treated in serumfree DMEM containing 2 mM glucose in normoxia or hypoxia (0.1%) for 22 h. Cell death was analyzed by PI staining and quantified by flow cytometry (*n* = 4, mean ± SEM, student’s t-test/two-way ANOVA followed by Tukey’s multiple comparisons test, *p < 0.05, **p < 0.01, ***p < 0.001). **G** NCH 690 SOD1sh cells were treated in serumfree DMEM containing 2 mM glucose, 20 ng/ml bFEF and 20 ng/ml EGF in normoxia or hypoxia (0.1%) for 18 h. LN-229 *SOD1* KO cells were treated in serumfree DMEM containing 2 mM glucose in normoxia or hypoxia (0.1%) for 14 h. After these timepoints NADP-NADPH-Glo-Assay was performed and cells were analyzed by NADPH/NADP ratio (*n* = 3, mean ± SEM, student’s t-test, *p < 0.05, **p < 0.01, ***p < 0.001).

To confirm the robustness of the observed phenotype *SOD1* knockdown was performed in primary glioma cells NCH 690 and a *SOD1* CRISPR/Cas9 knockout was performed in LN-229 cells. Western blot analysis confirmed knockdown/knockout efficiency (Fig. 4A). SOD activity was analyzed using SOD activity assay (Fig. 4B). Under starvation conditions these cells were also characterized by an increase in ROS levels (Fig. 4C). In addition, growth of knockout cells was restricted after four days for Clone # 3 (Fig. 4D) and clonogenic survival was inhibited (Fig. 4E). Measurements of PI uptake confirmed the results of an increased sensitivity to hypoxia-induced cell death (Fig. 4F). Additionally, the NADPH/NADP ratio was reduced in *SOD1* knockdown/knockout cells (Fig. 4G).

### The small molecule inhibitor ATN-224 sensitizes GB cells to starvation conditions

**Fig. 5:**
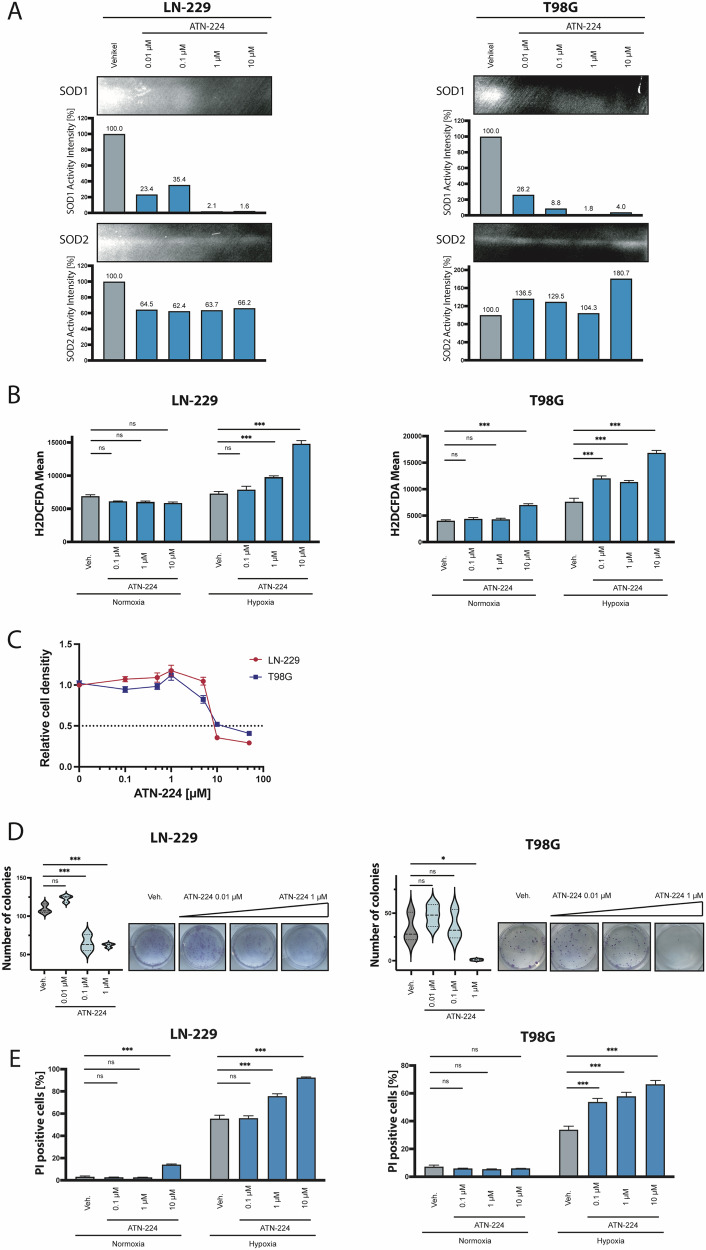
The small molecule inhibitor ATN-224 inhibits SOD1 activity, sensitizes GB cells for hypoxia-induced cell death and the combination with rapamycin abrogates the protective effect of mTORC1 monotherapy. **A** LN-229 and T98G cells were treated with the SOD1 inhibitor ATN-224 in serumfree DMEM and analyzed by SOD1/2 enzyme activity assay. ATN-224 treatment decreased SOD1 activity up to 99%. **B** LN-229 and T98G cells were preincubated with ATN-224 for 24 h in serumfree DMEM containing 25 mM glucose. Afterwards cells were treated with the SOD1 inhibitor ATN-224 in serumfree DMEM containing 2 mM glucose in normoxia or hypoxia (0.1%) for 25 h (LN-229) and 16 h (T98G). Cells were analyzed by H2DCFDA and PI staining. At this timepoint no relevant cell death was observed (data not shown) and only PI negative ( = living) cells were quantified for H2DCFDA FACS analysis (*n* = 4, mean ± SEM, two-way ANOVA followed by Tukey’s multiple comparisons test, *p < 0.05, **p < 0.01, ***p < 0.001). **C** LN-229 and T98G cells were treated with the SOD1 inhibitor ATN-224 (Veh. (DMSO), 0.1 µM, 0.5 µM, 1 µM, 5 µM, 10 µM, 20 µM) in serumfree DMEM in normoxia for 96 h. Cell density was analyzed by CV staining. **D** LN-229 and T98G cells were seeded (500 cells per well) in 6-well-plates and after 24 h treated with the SOD1 inhibitor ATN-224 with a concentration of 0.01 µM, 0.1 µM and 1 µM. Cell growth of the clones was stopped via CV staining after 10 days (*n* = 3, one-way ANOVA followed by Tukey’s multiple comparisons test, *p < 0.05, **p < 0.01, ***p < 0.001). **E** LN-229 and T98G cells were preincubated with ATN-224 for 24 h in serumfree DMEM containing 25 mM glucose. Afterwards cells were treated with the SOD1 inhibitor ATN-224 in serumfree DMEM containing 2 mM glucose in normoxia or hypoxia (0.1%) for 22 h (LN-229) and 10 h (T98G). Cells were analyzed by PI staining (*n* = 4, mean ± SEM, two-way ANOVA followed by Tukey’s multiple comparisons, *p < 0.05, **p < 0.01, ***p < 0.001).

To confirm the observed genetic effects pharmacological SOD1 inhibition using the small molecule SOD1 inhibitor ATN-224 was performed [36]. ATN-224 has not been investigated in glioma cells so far. Different concentrations of the inhibitor (0.01 µM to 10 µM) were tested in LN-229 and T98G (Fig. 5A) glioma cells using the SOD1/2 activity assay [37]. In both cell lines, considerable effects regarding SOD1 activity could already be observed with a concentration of 0.01 µM. Concentrations of 1 µM ATN-224 led to a SOD1 inhibition up to 98%. ROS level of LN-229 and T98G glioma cells after treatment with ATN-224 were examined under nutrient deprivation and hypoxia. Both cell lines showed a dose-dependent increase in ROS levels under hypoxic conditions, starting at an ATN-224 concentration of 0.1 µM (Fig. 5B). Under normoxic conditions only 10 µM ATN-224 led to an increase in ROS in T98G cells (Fig. 5B). Cell density after ATN-224 treatment under standard culture conditions was relevant decreased only at higher concentrations starting with 10 µM (Fig. 5C). In contrast, ATN-224 showed a significant reduction in clonal growth at lower concentration of 0.1 µM or 1 µM depending on the tested cell line (Fig. 5D). Cell death analysis confirmed an increased sensitivity to hypoxia-induced cell death for ATN-224 concentrations of 0.1 µM and higher (Fig. 5E).

### mTORC1-dependent regulation of SOD1 as a possible explanation for the resistance of GBs to mTORC1 inhibitors

**Fig. 6:**
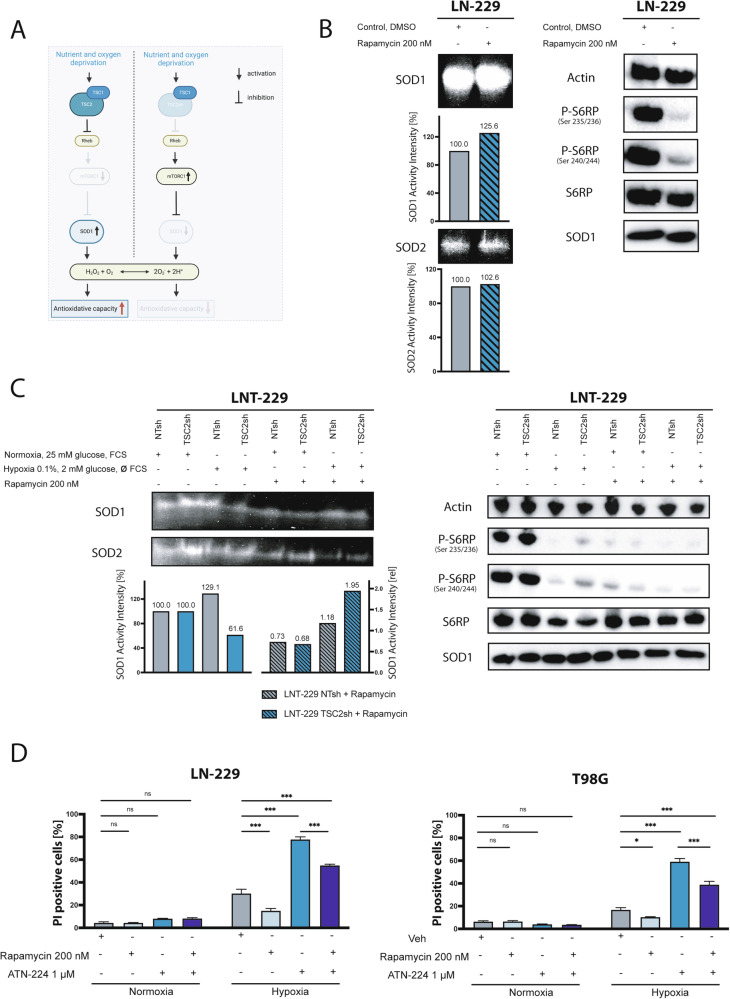
The mTORC1 dependent SOD1 regulation as a possible explanation for the resistance of GBs to mTORC1 inhibitors. **A** Under nutrient and oxygen deprivation *TSC2* knockdown leads to activation of mTORC1, which inhibits SOD1 activity via phosphorylation (Fig. 5A right panel, Created with BioRender.com). **B** LN-229 were treated for 4 h with or without rapamycin in serumfree DMEM containing 25 mM glucose and the growth factors EGF and FGF2. SOD1 and SOD2 enzyme activity were analyzed by enzyme activity assay showing an increase in SOD1 activity. Effective mTORC1 inhibition with rapamycin was proven by reduced mTORC1 activity (P-S6RP (Ser 235/236 and Ser 240/244)) in western blot analysis. **C** LNT-229 TSC2sh and control cells (NTsh) were treated for 4 h with serum containing DMEM with 25 mM glucose in normoxia or under starvations conditions with serumfree DMEM with 2 mM glucose in hypoxia (0.1%) with or without rapamycin. SOD1 and SOD2 enzyme activity was analyzed by enzyme activity assay showing a decrease in SOD1 activity in TSC2sh cells under starvation. SOD1 activity of all rapamycin-treated cells was analyzed relative to their non-rapamycin-treated counterparts (striped bars). mTORC1 activity was analyzed by western blot of downstream target proteins showing an enhanced mTORC1 activity (P-S6RP (Ser 235/236 and Ser 240/244)) of TSC2sh cells under starvation compared to control cells and an effective mTORC1 inhibition with rapamycin. **D** LN-229 and T98G cells were preincubated +/- ATN-224 for 48 h in serumfree DMEM containing 25 mM glucose. In case of treatment with the mTORC1-inhibitor, after 24 h rapamycin was added for another 24 h in serumfree DMEM containing 25 mM glucose. After the preincubation, cells were treated in serumfree DMEM containing 2 mM glucose +/- ATN-224/rapamycin in normoxia or hypoxia (0.1%) for 27 h (LN-229) or 12 h (T98G). Cells were analyzed by PI staining and quantified by FACS analysis (*n* = 4, mean ± SEM, two-way ANOVA followed by Tukey’s multiple comparisons, *p < 0.05, **p < 0.01, ***p < 0.001).

The results of *SOD1* knockdown/knockout and the pharmacological SOD1 inhibition using ATN-224 demonstrated a significant influence of SOD1 on the adaptation of glioma cells to the tumor microenvironment. Therefore, we further investigated the influence of the mTORC1 pathway on the regulation of SOD1 activity under starvation conditions. We hypothesized that under nutrient and oxygen deprivation *TSC2* knockdown cells display an activated mTORC1 signaling leading to an inhibition of SOD1 activity via phosphorylation in GB cells (Fig. 6A).

To study the mTORC1-SOD1 axis, SOD1 activity was analyzed after treatment with the mTORC1 inhibitor rapamycin and vice versa in mTORC1-activated *TSC2* knockdown cells (TSC2sh) [29]. mTORC1 as well as SOD1 enzyme activity were analyzed under hypoxia and nutrient deprivation. Treatment of glioma cells with rapamycin enhanced SOD1 activity (Fig. 6B). Inhibition of mTORC1 signaling was confirmed by Western blot analysis (Fig. 6B). Knockdown of *TSC2* in LNT-229 and LN-308 cells resulted in activation of mTORC1 downstream signaling under starvation conditions as already described [29] and was accompanied by a reduced SOD1 activity (Supplementary Fig. 2A, B, Fig. 6C).

An additional rescue experiment to validate the mTORC1-dependent regulation of SOD1 was performed: The decrease in SOD1 activity in mTORC1 activated TSC2sh cells under deficiency conditions was rescued using the mTORC1 inhibitor rapamycin which induced a relative increase in SOD1 activity (Fig. 6C). These findings demonstrate the mTORC1-dependent regulation of SOD1 in GB cells.

To test the hypothesis weather SOD1 activation could be a potential explanation for the known therapy resistance of GBs to mTORC1 inhibitors under conditions of the tumor microenvironment [25, 26], the combination therapy of the mTORC1 inhibitor rapamycin and the SOD1 inhibitor ATN-224 was tested under starvation conditions. Rapamycin monotherapy conferred protection against hypoxia-induced cell death as described previously [25, 26]. In contrast, ATN-224 monotherapy increased cell death (Fig. 6D). Combination therapy with rapamycin and ATN-224 showed higher cell death under hypoxia compared to rapamycin monotherapy, but also lower cell death than the ATN-224 monotherapy (Fig. 6D). Therefore, ATN-224 at least partially antagonized the protective effects of mTORC1 inhibition under conditions of the glioma microenvironment.

## Discussion

In this study, we identify SOD1 as an important factor enhancing cell survival under conditions of the GB microenvironment and as a potential mediator of therapy resistance. We have included energy-limiting conditions mirroring the glioma microenvironment to analyze mechanisms of starvation induced mTORC1 inhibition. In previous studies, we had already shown that pharmacological inhibition of mTORC1 triggers metabolic changes that cause negative effects by protecting hypoxic tumor areas from hypoxia-induced cell death [25, 38]. Hence, adaptive mTORC1 inhibition is a possible mechanism of intrinsic resistance to microenvironmental conditions, starvation-inducing therapies and may be enhanced by pharmacological inhibition of mTORC1. A possible explanation for this resistance mechanism could be the activation of superoxide dismutase 1, especially in regions with low nutrient and oxygen conditions. In our study, we examined the role of the mTORC1-SOD1 axis and observed a significant decrease of SOD1 enzyme activity following mTORC1 activation. Our results indicate that activation of SOD1 contributes to mTORC1 inhibition-mediated protective effects in the tumor microenvironment [25, 39, 40]. In line with this hypothesis, vice versa *SOD1* knockdown/knockout increased ROS levels and sensitized to nutrient deprivation and hypoxia.

Our results on the role of SOD1 for hypoxia-induced cell death are in line with previous reports showing the importance of SOD1 for cell survival and redox homeostasis under starvation conditions [14]. Understanding the dual role of ROS in cancer is crucial for the understanding of cancer intrinsic therapy resistance. Cancer cells are characterized by per se higher basal ROS levels [21]. At lower concentrations ROS can act as signaling molecules for procarcinogenic effects including proliferation, invasion and angiogenesis. On the other hand, extensive ROS can lead to cell death by causing damage to cellular components including proteins and organelles [21, 41, 42]. Our mechanistic studies indicated that enhanced SOD1 activity protects GB cells from conditions of the glioma microenvironment by maintaining redox homeostasis. In line, inhibition of SOD1 resulted in an imbalance of the NADPH/NADP ratio resulting in a higher susceptibility to redox stress and reduced survival under starvation conditions. Therefore, induction of SOD1 activity via mTORC1 seems to be one resistance mechanism under starvation conditions. Similarly, it has been observed that upregulation of serine and glycine metabolism seem to be part of the adaptive processes involved in metabolic adaptation to starved tumor regions. It has already been shown that serine *hydroxymethyltransferase 2 (SHMT2)* was highly expressed in glioma cells surrounding the necrosis zone and hypoxia drives the expression of the key enzymes of serine metabolism in glioma [9].

For the first time we could demonstrate that SOD1 inhibition in glioma cells using the small molecule inhibitor ATN-224 sensitizes GB cells towards conditions of the tumor microenvironment including glucose and oxygen deprivation. Although the combination therapy of the mTORC1 inhibitor rapamycin and the SOD1 inhibitor ATN-224 failed to achieve increased cell death rates in our study compared to SOD1 inhibitor monotherapy, the protective effect of the mTORC1 inhibitor rapamycin was reversed to some extent, and potential benefits of this combination therapy should be further investigated.

One aspect limiting the application of a SOD1 inhibitor in cancer could be toxicity. Remarkably, a genetic whole-body deficiency of *SOD1* only led to spontaneous nodular liver hyperplasia or hepatocellular carcinoma by 20 months of age [43] which could be caused by the capacity of normal cells to switch to alternative ways of maintaining redox homeostasis. It is already known that SOD1 inhibition via LCS-1 induces ROS-mediated cell death, which could be caused by the failure of DNA repair mechanisms [31].

Taken together, SOD1 seems to be a plausible target for therapeutic inhibition in GBs. Even though we demonstrated that gene suppression of *SOD1* in glioma cells has significant effects on cell survival, further in vivo animal studies and clinical trials on the efficacy and safety of ATN-224 monotherapy and combinational therapy with e.g. irradiation or mTORC1 inhibitors are exciting future options for a SOD1-targeted therapeutic approach to GBs. In some other tumor entities (including esophageal cancer, prostate cancer or lung metastases), the inhibitor and its active ingredient tetrathiomolybdate have already been tested in clinical trials and provided important findings for potential clinical trials in glioblastoma patients [44–46]. Advantages are the oral application and the good tolerability [44–47]. With regard to tumor subgroups, it is plausible that SOD1 inhibition might be especially effective in tumors with an intact mTORC1 sensor, i.e. tumors that effectively downregulate mTORC1 signaling in response to hypoxia and/or nutrient deprivation or in combination with mTORC1 inhibitors. Biomarkers to determine mTORC1 signaling in tumor tissue [48] and to identify GBs that might benefit from a targeted therapy have been established [49, 50].

## Materials/Subjects and Methods

### Expression and survival analyses

Expression and survival analyses were performed on the Gliovis website using the TCGA GBM and Rembrandt databases [35].

### Reagents, cell lines and culture conditions

All reagents not specified were purchased from Sigma (Taufkirchen, Germany). LN-229, T98G, U-118MG and LN-18 human malignant glioma cell lines were purchased from ATCC. LNT-229, LN-308 and A172 cells were a kind gift of Dr. N. de Tribolet (Lausanne, Switzerland) [25, 51, 52]. LNT-229/LN-229, T98G, LN-308 and NCH 690 cells were authenticated using MCA or STR analysis by Multiplexion (Heidelberg, Germany). The STR profile of the tested cells matched with the known profile for LN-229. G55T2 cells were a kind gift of Manfred Westphal and Kathrin Lamszus (Hamburg, Germany) [53], LN-428 and LN-464 cells were a kind gift from Monika Hegi (Lausanne). NCH 690, NCH 644 and NCH 421 K primary glioma cells were purchased from CLS (Eppelheim, Germany) [54]. ATN-224 was purchased from Cayman Chemical (Cay23553-5). LN-229, T98G, LNT-229, LN-308, A172, G55T2, U-118MG, LN-18, LN-428 and LN-464 cells were maintained in Dulbecco’s modified eagle medium (DMEM) containing 10% fetal calf serum (FCS) (Biochrom KG), 100 IU/ml penicillin and 100 µg/ml streptomycin (Life Technologies). NCH 690, NCH 644 and NCH 421 K cells were cultured in Neurobasal A medium supplemented with 2% glutamine, B27 supplement, 1 U/ml heparin, 20 ng/ml human recombinant basic fibroblast (bFEF), 20 ng/ml epidermal growth factor (EGF) (ReliaTech, Wolfenbüttel, Germany) and 100 IU/ml penicillin and 100 µg/ml streptomycin (Life Technologies, Karlsruhe, Germany). pLKO.1 transfected cells were additionally maintained in medium containing 2 µg/ml puromycin. Human astrocytes were purchased from Innoprot (Derio, Spain) and were cultured in Astrocyte medium (Innoprot, Derio, Spain) according to the manufacturer’s protocol.

### Generation of *SOD1* and *TSC2* knockdown cells

The pLKO.1 plasmid (Sigma, Clone-ID: TRCN 00000 18344 (SOD1sh Seq1), TRCN 0000039808 (SOD1sh Seq2) and TRCN 00000 39812 (SOD1sh Seq3)) were used to induce stable shRNA-mediated gene suppression of *SOD1*. Control cells were transfected with a pLKO.1 plasmid with a non-targeting shRNA sequence (NTsh) (Addgene catalog no. 1864, Watertown, MA, USA). Polybrene (Millipore) was used to facilitate lentiviral transduction with a virus concentration corresponding to a MOI of 80. The MOI was determined using the Lenti-X p24 Rapid Titer Kit (Takara Bio). *SOD1* expression was quantified by qPCR and western blot analysis. *TSC2* knockdown cells (TSC2sh) have already been described [29]. For selection and cultivation 2 µg/ml puromycin (Sigma) was added to the culture medium.

### Generation of *SOD1* CRISPR/Cas9 knockout cells

*SOD1* knockout cells were created by using CRISPR/Cas9 sgRNA plasmids targeting exon 1 and exon 4 of the *SOD1* gene (pSpCas9 BB-2A-Puro (pX459) V2.0 9.2 kb, Plasmid #62988, from Genscript, Rijswijk, Netherlands, gRNA 1 TTGCATCATTGGCCGCACAC and gRNA 5 AGCATTAAAGGACTGACTGA). LN-229 cells were transfected with the gRNA plasmids (1 µg each) using Lipofectamine3000 (Thermo Fisher Scientific, Hamburg, Germany) and incubated for 48 h. Afterwards cells were selected using puromycin 2 µg/ml. Single cell clone analysis for SOD1 expression was performed via immunoblot.

### RNA extraction and quantitative reverse transcription-PCR analysis (RT-PCR)

The quantitative PCR analysis (qPCR) protocol has already been described [29]. Briefly, for RNA purification TRIzol and the RNeasy kit (Invitrogen) were used according to the manufacturer’s protocol. cDNA was synthesized using Vilo cDNA Synthesis Kit (Invitrogen) (10 min at 25 °C followed by 2 h at 42 °C) according to the manufacturer’s instructions. For qPCR, the IQ5 real-time PCR detection system (Bio-Rad) was performed using Absolute Blue qPCR Mastermix with SYBR Green (Thermo Fisher Scientific) and the following cycle parameters: Cycle 1 (95 °C for 15 min), Cycle 2 (95 °C for 15 s), Cycle 3 (58 °C for 30 s), Cycle 4 (72 °C for 30 s), repeat Cycle 2 to 4 for 39 times, Cycle 5 (95 °C for 30 s), Cycle 6 (Melt Curve 60 °C to 100 °C), Cycle 7 (4 °C forever).

The following primer pairs were used: *18* *S*: Fwd: 5’-CGGCTACCACATCCAAGGAA-3’, Rev: 5’-GCTGGAATTACCGCGGCT-3’; *Succinate dehydrogenase complex subunit A (SDHA)*: Fwd: 5’-TGGGAACAAGAGGGCATCTG-3’, Rev: 5’-CCACCACTGCATCAAATTCATG-3’; *SOD1*: Fwd: 5’-GGTGGGCCAAAGGATGAAGAG-3’, Rev: 5’-CCACAAGCCAAACGACTTCC-3’; *SOD2*: Fwd: 5’-GCTCCGGTTTTGGGGTATCTG-3’, Rev: 5’-GCGTTGATGTGAGGTTCCAG-3’; *SOD3*: Fwd: 5’-ATGCTGGCGCTACTGTGTTC-3’, Rev: 5’-CTCCGCCGAGTCAGAGTTG-3’.

For normalization, 18 S and SDHA were both used as housekeeping genes. Analysis was performed according to the Vandesompele method [55].

### Induction of hypoxia

Induction of hypoxia was carried out as previously described [25, 38, 56]. Briefly, hypoxic conditions of 0.1% oxygen were induced by incubation in GasPak^TM^ pouches for anaerobic culture (Becton-Dickinson, Heidelberg, Germany) in serum-free DMEM adjusted to 2 mM glucose.

### Cell density and cell viability assay

Cell growth assays were performed in 96-well plates. Cells were seeded 24 h before treatment and quantified by crystal violet (CV) after the appropriate incubation period. Cell death analysis was performed by lactate dehydrogenase (LDH) release assay and via propidium iodide (PI) uptake in flow cytometry as previously described [26]. When comparing different subcell lines, equal cell densities were confirmed using crystal violet staining in a parallel assay [25, 29, 57].

### Measurement of reactive oxygen species

For analysis of reactive oxygen species (ROS), cells were seeded in 24-well plates 24 h before treatment. Cells were analyzed by H2DCFDA and PI staining. At this timepoint no relevant cell death was observed and only PI negative ( = living) cells were quantified for H2DCFDA (Invitrogen, catalog no. D399) flow cytometry analysis.

### SOD activity assay

SOD 1/2 activity was determined by in-gel activity assay published by Weydert et al. [37] with only slight modifications (separating gel was produced using 235 µl 10% APS instead of 68 µl and 10 µl TEMED instead of 9 µl). Cell lysates for SOD activity assay were prepared as described by Tsang et al. [14]. The quantification tool of Image Lab 6.1 (Bio-Rad Laboratories, Inc., California, USA) was used to quantify the lanes and bands.

### Immunoblot

Immunoblot was performed as already described [29, 58]. Briefly, cells were washed with ice-cold PBS after incubation and frozen by fluid nitrogen. Cell lysates were prepared using lysis buffer P and protein content was determined by Bradford analysis. For SDS Page analysis, 10 µg protein were applied. After blocking in 5% milk, membranes were placed in antibodies against SOD1 (Santa Cruz Biotechnology, Dallas, TX, USA, #sc-515404 C-8, working concentration 1:1000), SOD2 (Merck Sigma-Aldrich, Darmstadt, Germany, #06-984, 1:1000), Actin (Santa Cruz Biotechnology, Dallas, TX, USA, #sc-1616, 1:500), Actin (Abcam Limited, Cambridge, UK, #ab6276, 1:10000), TSC2 (Cell Signaling, Cambridge, UK, #43085, 1:2000), S6RP (Cell Signaling, #2217 s, 1:1000), P-S6RP (Ser 235/236, Cell Signaling, #4858 s, 1:2000), P-S6RP (Ser 240/244, Cell Signaling, #5364, 1:1000), 4-EB-P1 (Cell Signaling, #9452, 1:1000), P-4E-BP1 (Ser 65, Cell Signaling, #2855, 1:1000) overnight. Next day, membranes were incubated with secondary anti-mouse (Santa Cruz Biotechnology, #sc-516102), anti-goat (Santa Cruz Biotechnology, #sc-2020) and anti-rabbit antibodies (Jackson ImmunoResearch, #111-035-144). For detection chemiluminescence solution containing 1 ml solution A (200 ml 0.1 M Tris-HCl pH 8.6, 50 mg luminol), 100 µl solution B (11 mg p-hydroxy coumaric acid, 10 ml DMSO) and 0.3 µl H2O2 (30%) were used. Detection of immunoblot was carried out with the ChemiDoc MP Imaging System. The quantification tool of Image Lab 6.1 (Bio-Rad Laboratories, Inc., California, USA) was used to quantify the lanes and bands.

### NADPH/NADP ratio

A luminescence-based assay (NADP/NADPH-Glo Assay Kit, Promega, Madison, WI, USA) was used for the measurement of NADPH and NADP. All steps were performed according to the manufacturer’s protocol and have been described [58].

### Clonogenic assay

For analysis of clonogenic survival 500 cells per well were seeded in a 6-well plate. For experiments using ATN-224 treatment with ATN-224 was started after overnight incubation. The assay was stopped by crystal violet staining as long as the individual colonies could still be differentiated from each other.

### Statistical analysis

All data are expressed as mean ± SEM. For comparing two pairs of means statistical analysis was performed using the student’s t-test. Values of *p* < 0.05 were considered statistically significant, values of *p* < 0.01 were considered statistically very significant, and values of *p* < 0.001 were considered statistically extremely significant (Excel, Microsoft, Seattle, WA, USA).

For comparing three or more columns in grouped analysis with only one condition of data, statistical analysis was performed using the Shapiro-Wilk test to test normal distribution. In case of normal distribution, one-way ANOVA followed by Tukey’s multiple comparisons test was used to perform multiple comparison of the *p*-values. Values of *p* < 0.05 were considered statistically significant, values of *p* < 0.01 were considered statistically very significant, and values of *p* < 0.001 were considered statistically extremely significant (GraphPad (GraphPad Software, San Diego, CA, USA).

For comparing three or more columns in grouped analysis with two conditions of data (i.e. normoxia and hypoxia), statistical analysis was performed using the Shapiro-Wilk test to test normal distribution. In case of normal distribution, two-way ANOVA followed by Tukey’s multiple comparisons test was used to perform multiple comparison of the *p*-values. Values of *p* < 0.05 were considered statistically significant, values of *p* < 0.01 were considered statistically very significant, and values of *p* < 0.001 were considered statistically extremely significant (GraphPad (GraphPad Software, San Diego, CA, USA).

GraphPad (GraphPad Software, San Diego, CA, USA) was used to display the results.

### Supplementary information

Suppl. figures

Suppl. figure legend

Original western blots

## Data Availability

The corresponding author can provide the datasets used and/or analyzed during the current study upon reasonable request.
