# Supplementary figures and images for: Superoxide dismutase 1 mediates adaptation to the tumor microenvironment of glioma cells via mammalian target of rapamycin complex 1
Source: Cell Death Discov. 2024 Aug 26;10:379. doi: 10.1038/s41420-024-02145-6 (PMC11347576; doi:10.1038/s41420-024-02145-6)

# Suppl. Fig. 1

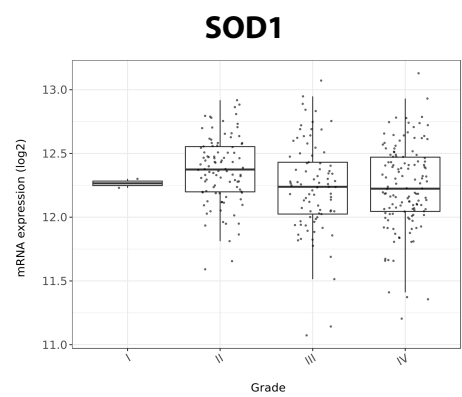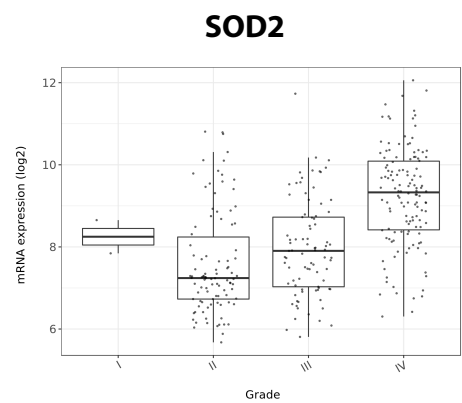

Suppl. Fig. 2

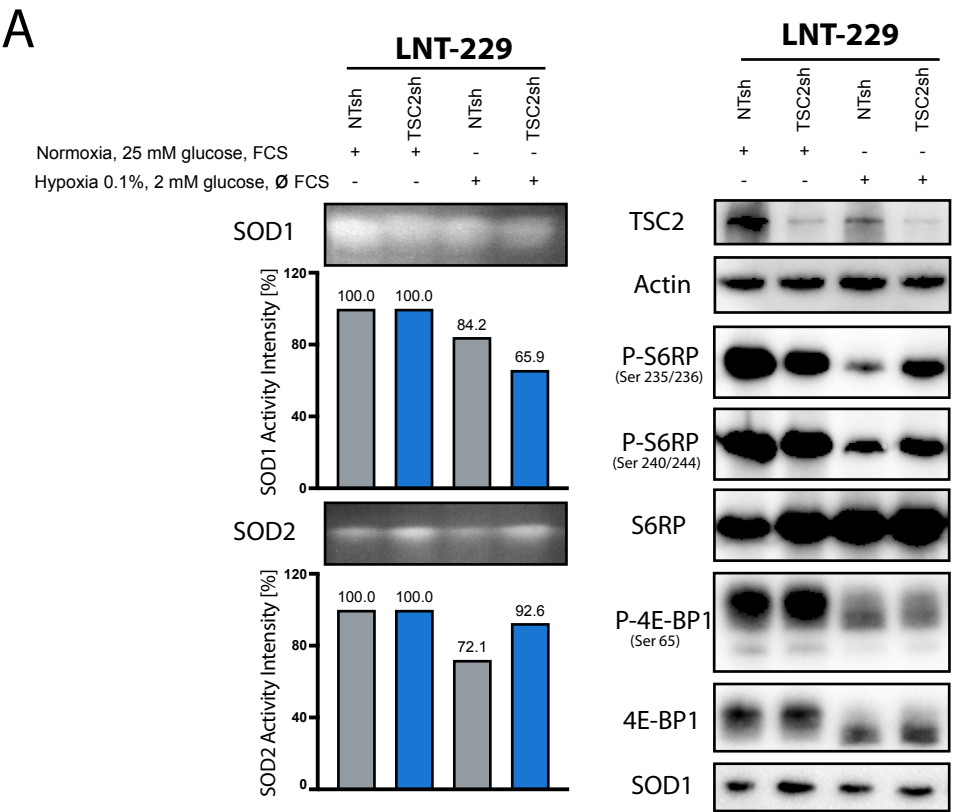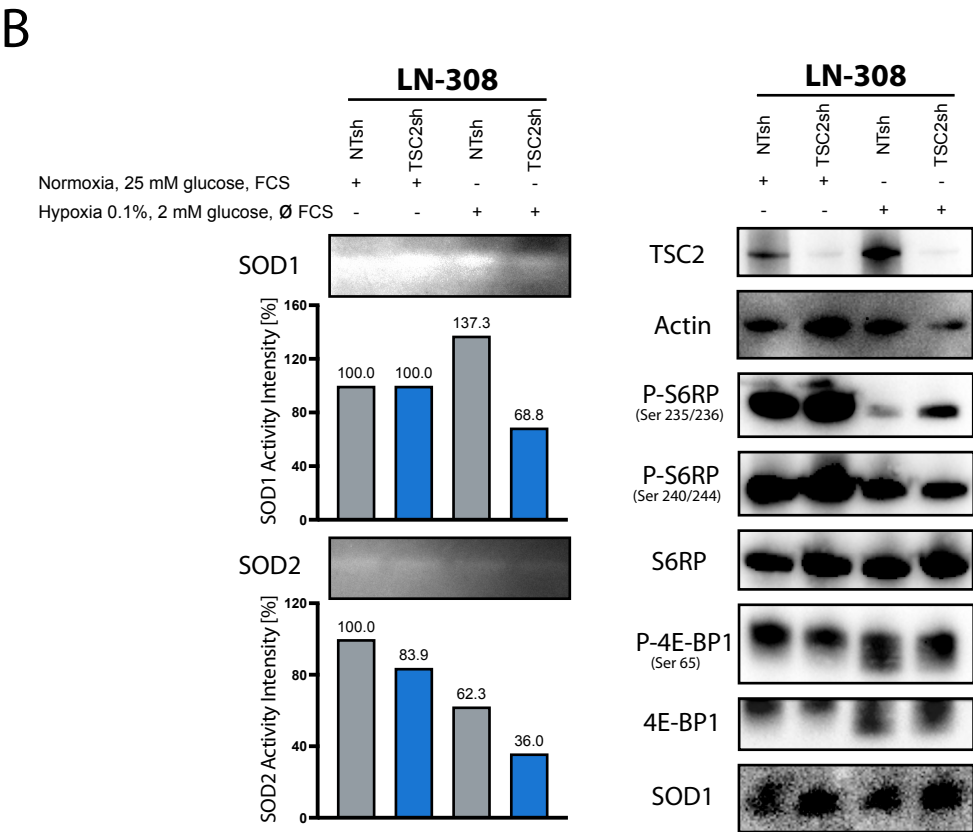

Supplement: Supplementary file 1 — Suppl. figures [file 41420_2024_2145_MOESM1_ESM.pdf]
