# Supplementary material for: Superoxide dismutase 1 mediates adaptation to the tumor microenvironment of glioma cells via mammalian target of rapamycin complex 1
Source: Cell Death Discov. 2024 Aug 26;10:379. doi: 10.1038/s41420-024-02145-6 (PMC11347576; doi:10.1038/s41420-024-02145-6)
Supplement: Supplementary file 2 — Suppl. figure legend [file 41420_2024_2145_MOESM2_ESM.pdf]

Suppl. figure legend

Suppl. Fig. 1: Analysis of SOD1 and SOD2 expression in gliomas of different WHO grades

Analysis of SOD1 and SOD2 expression in gliomas of different WHO grades using the Rembrandt database via Gliovis (ref. 31).

Suppl. Fig. 2: The mTORC1 dependent SOD1 regulation as a possible explanation for the resistance of GBs to mTORC1 inhibitors

(**A**) LNT-229 and (**B**) LN-308 TSC2sh and control cells (NTsh) were treated for 4 h with serum containing DMEM with 25mM glucose in normoxia or under starvations conditions with serumfree DMEM with 2mM glucose in hypoxia (0.1%). mTORC1 activity was analyzed by western blot of downstream target proteins showing an enhanced mTORC1 activity (P-S6RP (Ser 235/236 and Ser 240/244)) of TSC2sh cells under starvation compared to control cells. SOD1 and SOD2 enzyme activity was analyzed by enzyme activity assay showing a decrease in SOD1 activity in TSC2sh cells under starvation.
