# Supplementary material for: Superoxide dismutase 1 mediates adaptation to the tumor microenvironment of glioma cells via mammalian target of rapamycin complex 1
Source: Cell Death Discov. 2024 Aug 26;10:379. doi: 10.1038/s41420-024-02145-6 (PMC11347576; doi:10.1038/s41420-024-02145-6)

Original Western Blot

A (Fig. 1D)

SOD1

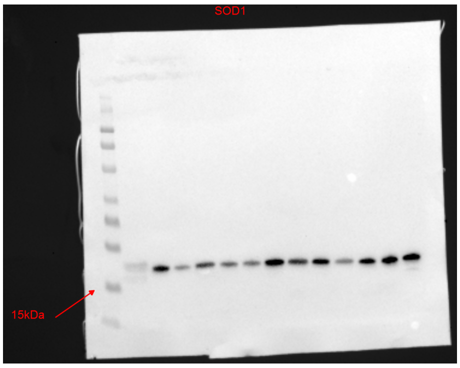

SOD2

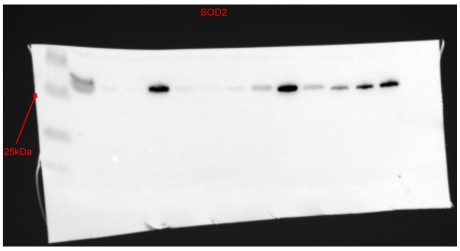

Actin

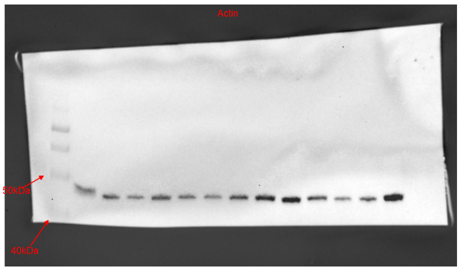

B (Fig. 2A, LN-229)

SOD1

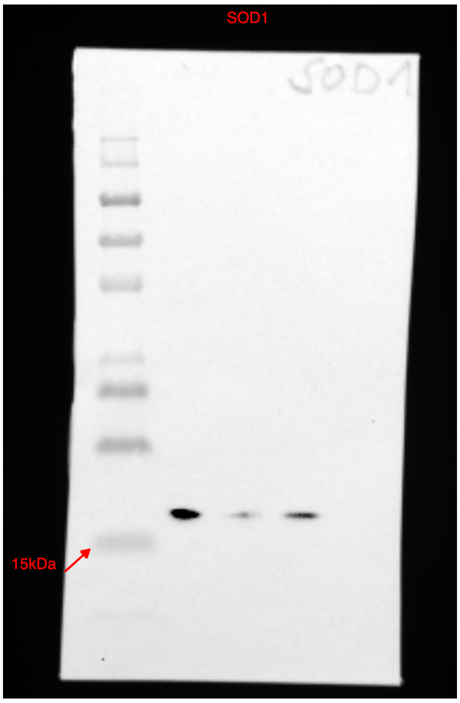

SOD2

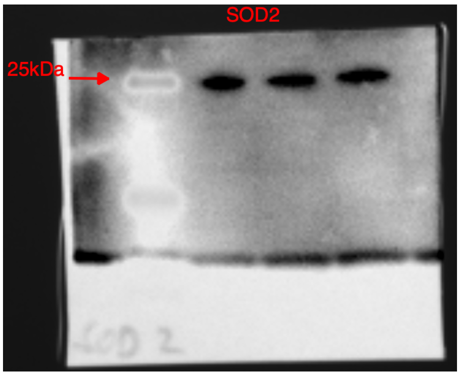

Actin

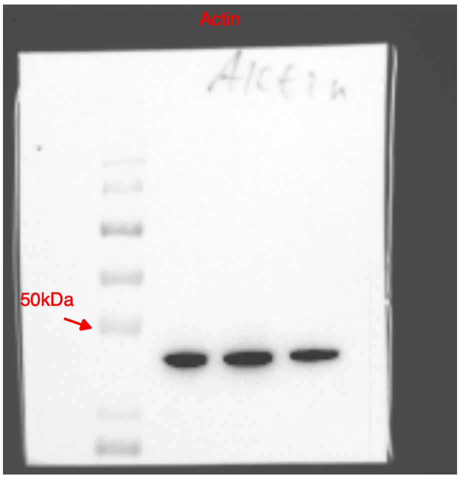

C (Fig. 2A, T98G)

SOD1

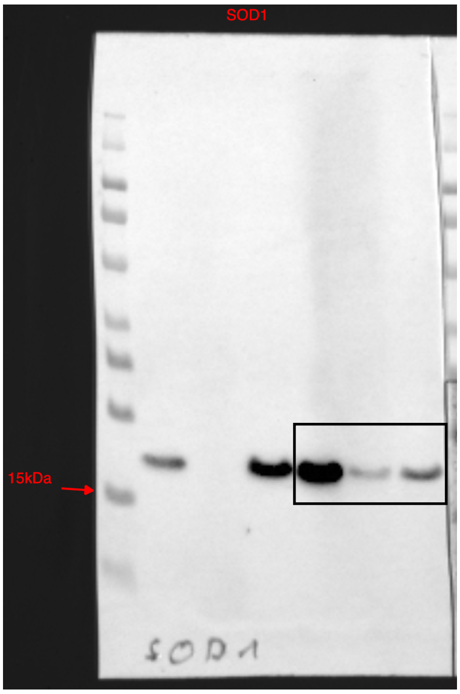

SOD2

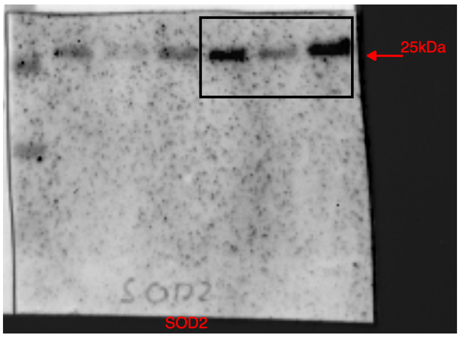

Actin

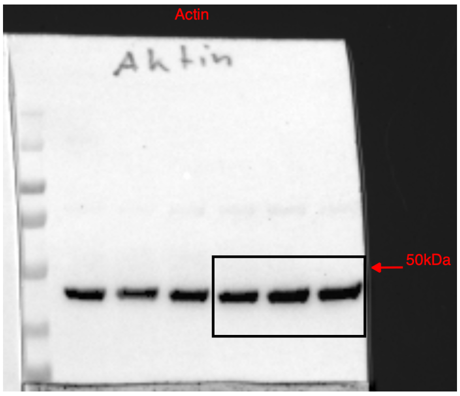

Original Western Blot

D (Fig. 4A, NCH 690)

SOD1

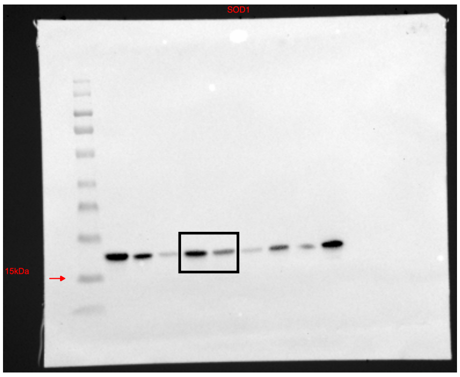

SOD2

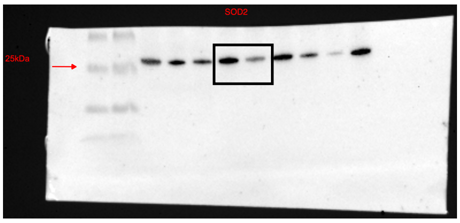

Actin

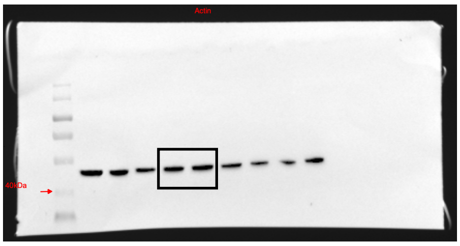

E (Fig. 4A, LN-229)

SOD1

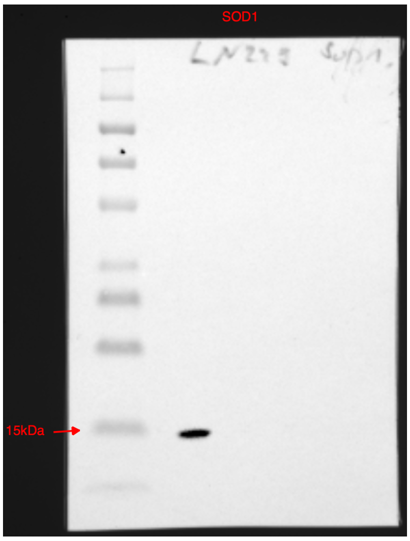

SOD2

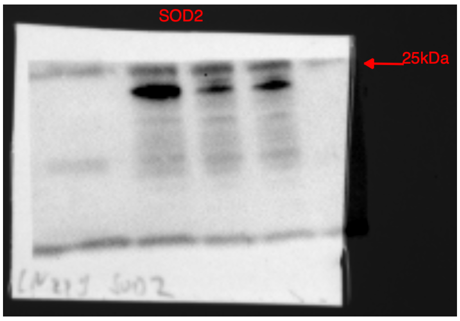

Actin

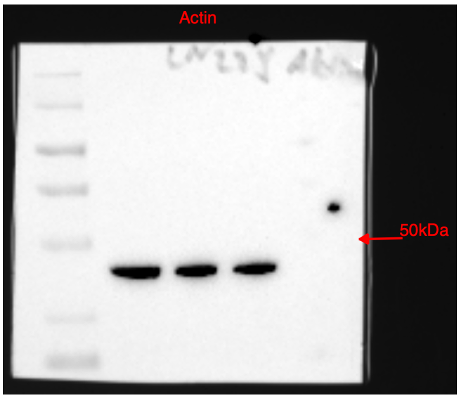

F (Fig. 6B, LN-229)

Actin

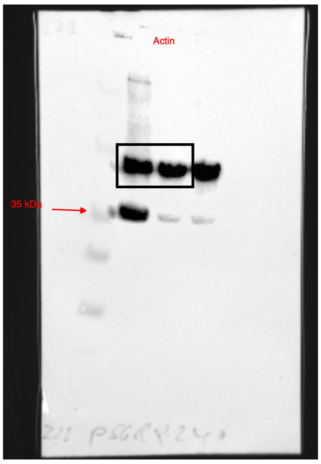

pS6RP (Ser 235/236)

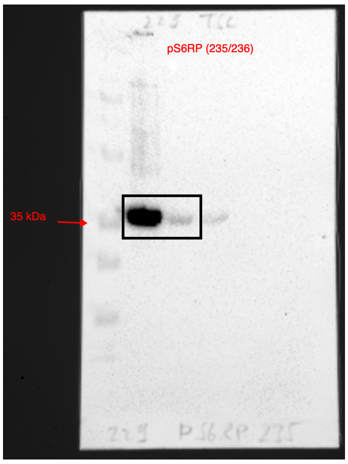

pS6RP (Ser 240/244)

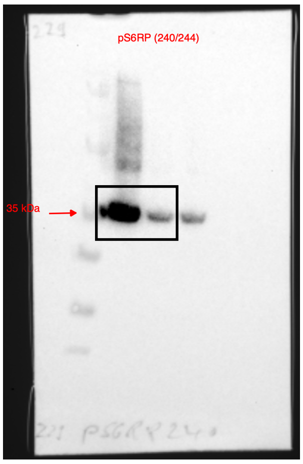

S6RP

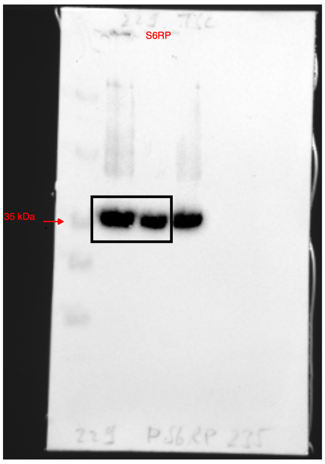

SOD1

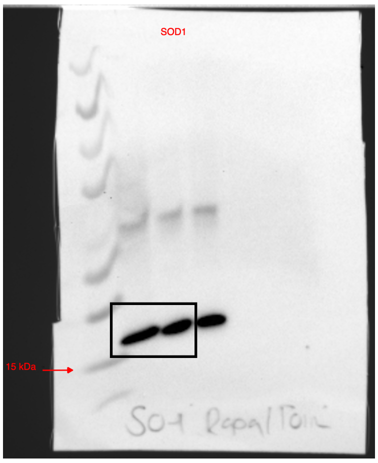

Original Western Blot

G (Fig. 6C, LNT-229)

Actin

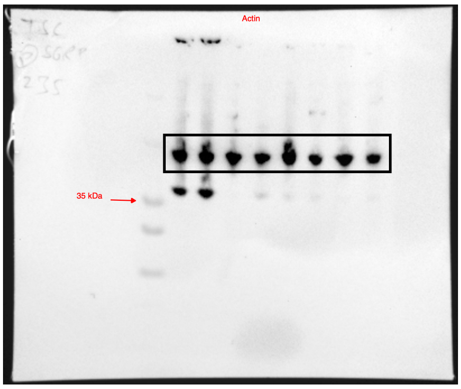

pS6RP (Ser 235/236)

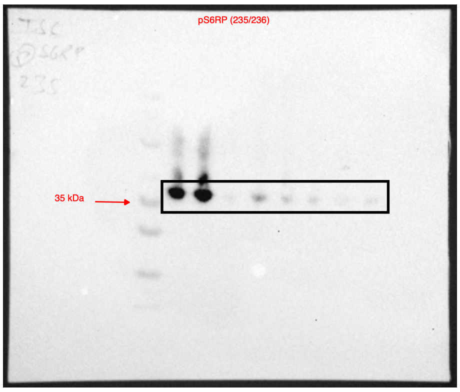

pS6RP (Ser 240/244)

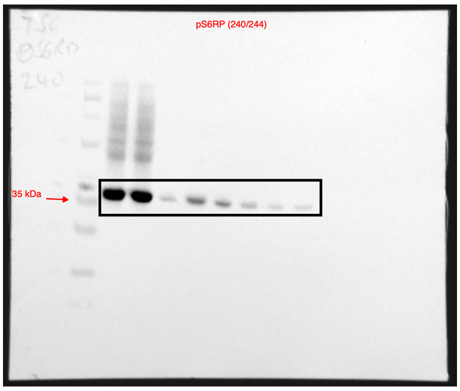

S6RP

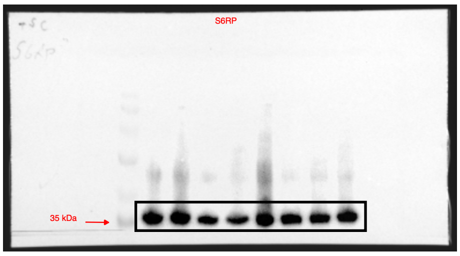

SOD1

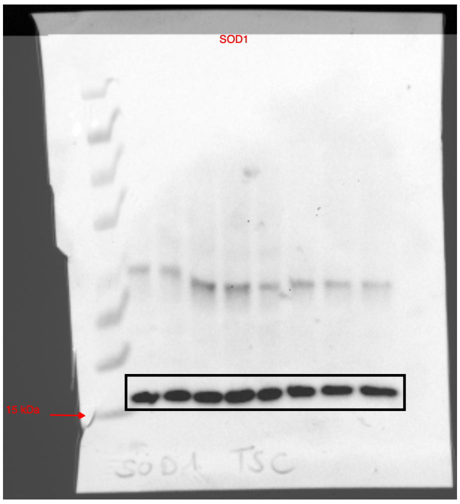

H (Suppl. Fig. 2A, LNT-229)

TSC2

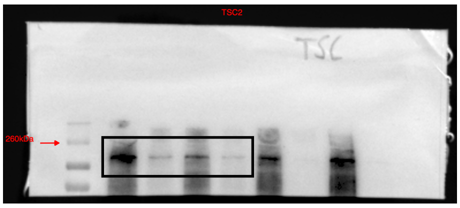

Actin

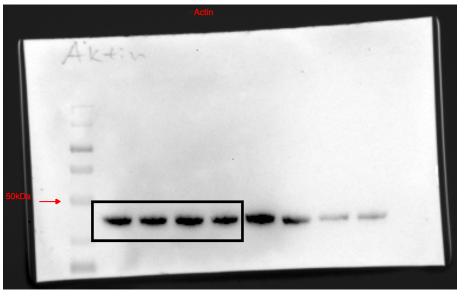

pS6RP (Ser 235/236)

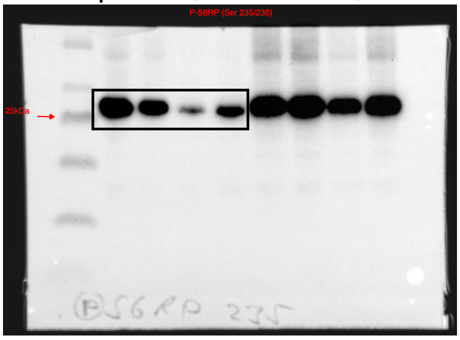

pS6RP (Ser 240/244)

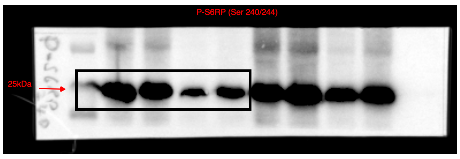

S6RP

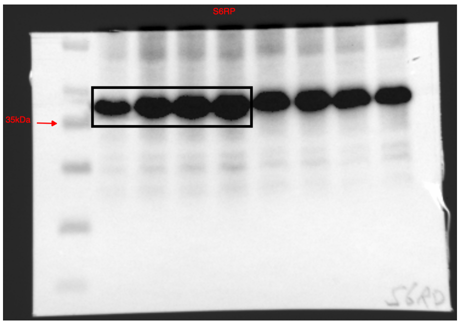

SOD1

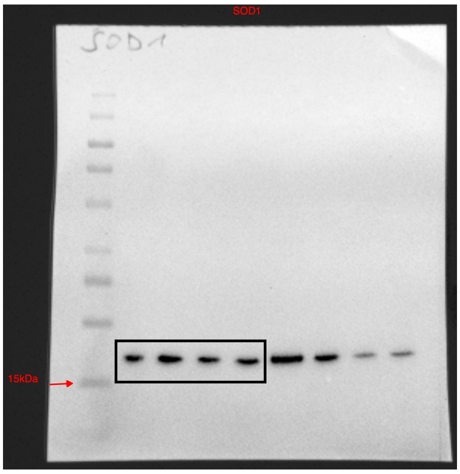

p4E-BP1 (Ser 65)

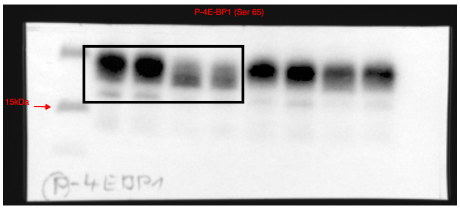

4E-BP1

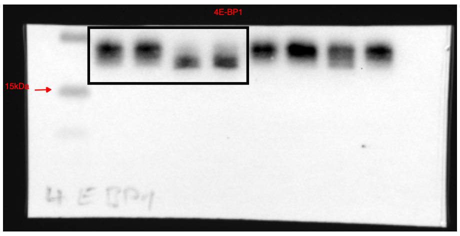

Original Western Blot

I (Suppl. Fig. 2B, LN-308)

TSC2

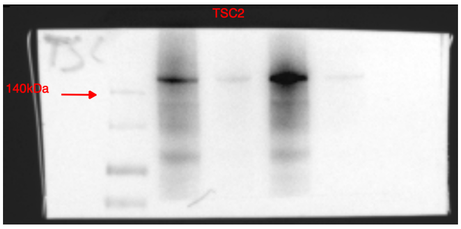

Actin

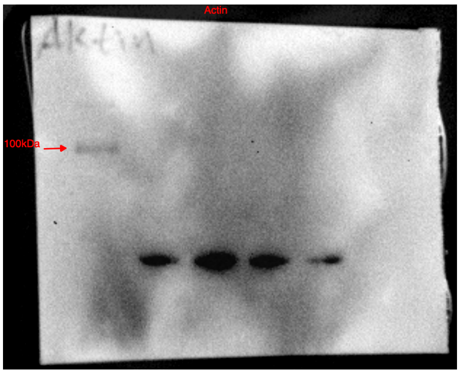

p-S6RP (Ser 235/236)

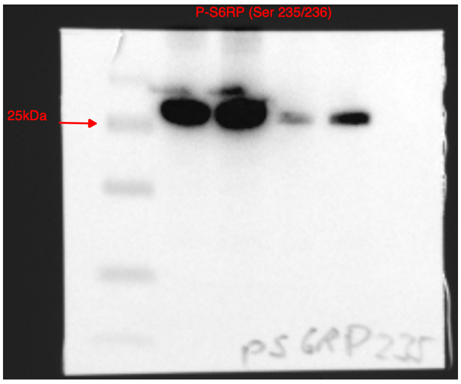

p-S6RP (Ser 240/244)

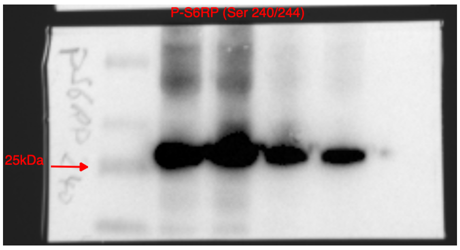

S6RP

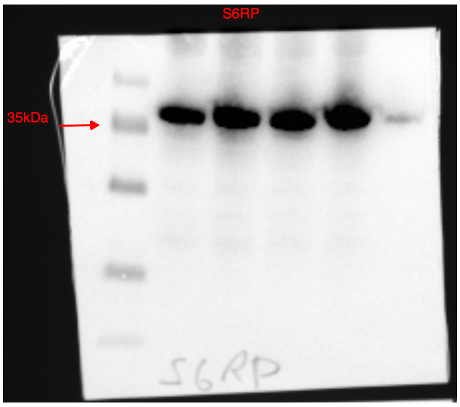

SOD1

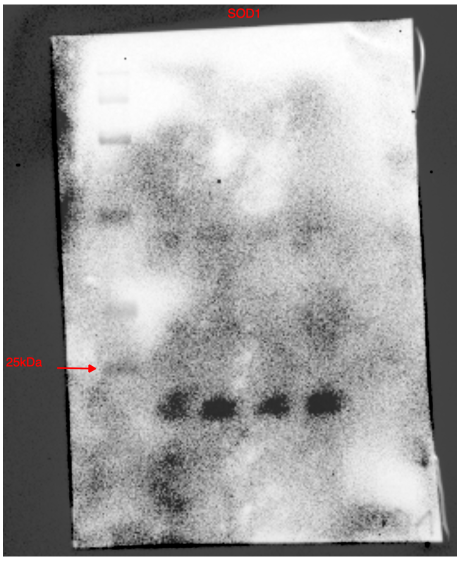

p-4E-BP1 (Ser 65)

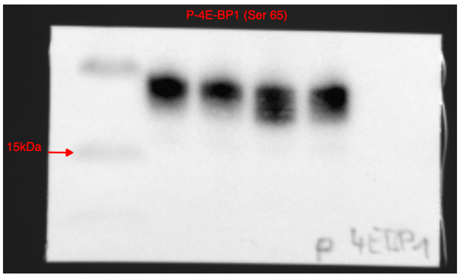

4E-BP1

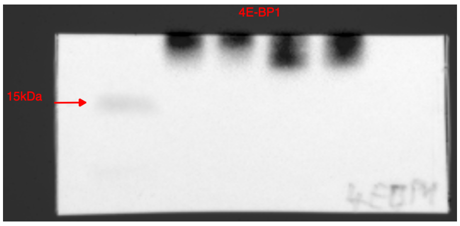

Supplement: Supplementary file 3 — Original western blots [file 41420_2024_2145_MOESM3_ESM.pdf]
